# Ankylosing Spondylitis: A rheumatology clinic experience

**DOI:** 10.12669/pjms.322.9366

**Published:** 2016

**Authors:** Tasnim Ahsan, Uzma Erum, Rukhshanda Jabeen, Danish Khowaja

**Affiliations:** 1 Prof. Tasnim Ahsan, MRCP (UK), FRCP(Glasg), FRCP(Edin), FRCP(Lon). Medical Unit-II, Rafiqee Shaheed Road, Jinnah Postgraduate Medical Centre, Karachi, Pakistan; 2 Dr. Uzma Erum, MBBS. Medical Unit-II, Rafiqee Shaheed Road, Jinnah Postgraduate Medical Centre, Karachi, Pakistan; 3 Dr. Rukhshanda Jabeen, MBBS, FCPS. Medical Unit-II, Rafiqee Shaheed Road, Jinnah Postgraduate Medical Centre, Karachi, Pakistan; 4 Dr. Danish Khowaja, MBBS. Medical Unit-II, Rafiqee Shaheed Road, Jinnah Postgraduate Medical Centre, Karachi, Pakistan

**Keywords:** Ankylosing Spondylitis, Spondyloarthropathy, Extra-articular features

## Abstract

**Objective:**

To determine the frequency, demographics, laboratory and radiological features in patients with Ankylosing Spondylitis.

**Methods:**

This is a retrospective analysis of prospectively collected data of patients with a diagnosis of Ankylosing Spondylitis (AS), based on Modified New York criteria. The study was conducted at the Rheumatology Clinic of Jinnah Postgraduate Medical Centre (JPMC), from February 2004 to February 2014. Detailed history, examination and laboratory investigations were recorded in a pre-designed structured proforma. The frequency, demographic characteristics, extra-articular features and associated co-morbidities were studied.

**Results:**

A total of 603 patients were registered in our Rheumatology Clinic during this period, with a definitive diagnosis of inflammatory rheumatological disorders. Out of these, Ankylosing Spondylitis (AS) was diagnosed in 32 (5.3%) patients. 24 were male and 8 patients were female. The commonest affected age group was between 21-40 years. Majority of the patients belonged to Pathan ethnicity.

**Conclusion:**

The demographic features of AS are same as reported in earlier studies from other parts of the world. The predominance of AS in specific ethnic groups is a fact that needs to be studied. Larger studies are required for clarifying the triggers of this disease. It often leads to severe disability, hence an early diagnosis and prompt treatment is required for better disease control and quality of life.

## INTRODUCTION

Spondyloarthropathies (SpA) encompass several rheumatic disorders that share clinical, genetic and radiographic features. Ankylosing Spondylitis (AS) is the prototype of SpA. It is a chronic, progressive, multi-system inflammatory disorder which primarily involves the sacroiliac (SI) joints and the axial skeleton. Peripheral joints and tendons can also be affected. The key to the diagnosis of AS is identifying features of inflammatory low back pain. Although, the aetiology of AS remains unclear, but there is evidence pointing to genetic predisposition. The human leucocyte antigen (HLA) B27 gene is commonly present. The prevalence of AS is variable, the disease is more common in Caucasians than in other races. Typically, the prevalence of AS in a population reflects the associated prevalence of HLA B27 gene in that population.^1^ A recent study reported a mean AS prevalence per 10,000 population as 23.8 cases in Europe, 16.7 in Asia, 31.9 in North America, 10.2 in Latin America and 7.4 in Africa.^2^ The exact prevalence, clinical and immunological aspects of AS in Pakistan, is largely unknown as studies pertaining to this are scarce. An approximate prevalence of SpA of 0.1 per 1000 population has been reported from Northern Pakistan.^3^ While, another study reported SpA prevalence of 0.9 per 1000 population from Southern part of the country.^4^

On the basis of the limited available data, there are no definite variables for the assessment of long-term outcome and mortality of AS patients. However, it has been reported that diagnostic delay, high CRP and work disability are the factors independently associated with reduced survival in these patients.^5^ Additionally, it is a disease of younger age group, affecting the capacity for gainful employment. Thus, AS patients have an important impact on health care and non health-care resource utilization as well. At present, there is no known curative treatment for AS. Goals of treatment are to reduce pain and stiffness, slow the progression of the disease, prevent deformity and preserve joint function and quality of life.

The objective was to determine the frequency, demographics, laboratory and radiological features in patients with Ankylosing Spondylitis.

## METHODS

### Study Design

This is a retrospective analysis of prospectively collected data of patients registered at the Rheumatology Clinic of Jinnah Postgraduate Medical Centre (JPMC) from February 2004 to February 2014. Modified New York criteria was used to diagnose AS. All patients with AS were registered. Data was recorded in a pre-designed structured proforma, that included history, examination, laboratory investigations, co-morbidities, treatment and follow-up records.

### Data Analysis

Data was analyzed by SPSS version-17. For descriptive statistics like gender, ethnic distribution, co-morbidities, extra-articular features, presence of HLA-B27, radiographic grading of Sacroiliitis and treatment regimen, frequency and percentages were calculated, while means were calculated for age and duration of joint pain and age of disease onset.

## RESULTS

**Fig.1:**
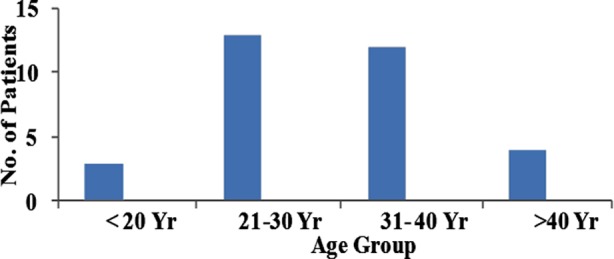
Age distribution of patients.

A total of 32 patients with definitive diagnosis of AS according to the Modified New York criteria, were included, which accounted for 5.3% of patients in the Rheumatology Clinic. Of them, 75% (n= 24) were male, and the rest (n=8) were female, with a male to female ratio of 3:1. The mean age of patients was 30.94±6.9 years, while the mean age of disease onset was 26.1±7.6 years. Most patients fell in the age group of 21-40 year, i.e. 25 patients, shown in Fig.1. The mean duration of joint pain was 4.9±3 years before presenting to our Rheumatology service. Out of the 32 patients, 27(84.4%) were positive for HLA-B27, while it was negative in two patients and was not checked in 3 patients.

**Fig.2:**
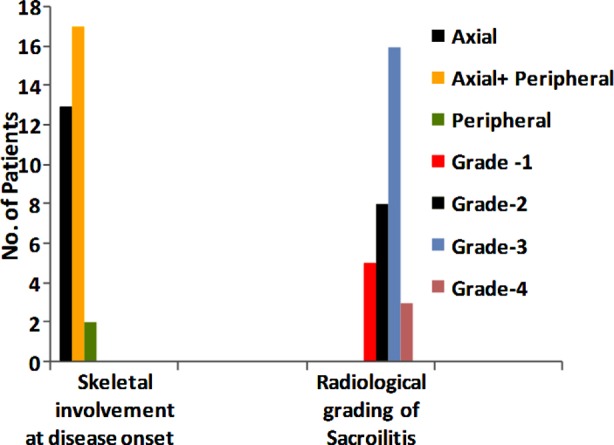
Skeletal involvement at disease onset and radiological grading of Sacroilitis.

The axial skeleton alone was involved in 13 (40.6%) patients; 2 (6.25%) had peripheral joint arthritis at disease onset, and rest of the patients had peripheral as well as axial joint involvement (Fig.2). Ethnic distribution showed that 16 patients (50%) belonged to Pathan ethnicity; 10 were immigrant Urdu-speaking (31.25%); 3 were Sindhi (9.37%); 2 were Punjabi (3.12%); and one was Egyptian. Radiologic grading of sacroiliitis showed grade-3 in 16 patients (50%) (Fig.2). None of the patients had any other unrelated co-morbidity. One patient had urethral discharge, one had episcleritis and 4 patients (12.5%) had fatigue as the major extra-articular symptom; rest had no extra-articular involvement. Amongst all, 19 patients (51.3%) received Sulphasalazine, 10 patients (31.25%) had NSAIDs alone, one patient (3.1%) was given Methotrexate, and two patients (6.2%) received anti-TNF (Etanercept).

## DISCUSSION

With the advent of new treatment modalities and better imaging technology, there has been major progress in the early diagnosis and management of AS in the last decade. AS is diagnosed in approximately 5% of patients with chronic low back pain.^6^ The prevalence of AS in a population is directly related to the frequency of HLA B27, thus the positive predictive value of HLA B27 is highest in populations with a low general prevalence for HLA B27. The association of HLA to the pathogenesis of AS might help in designing new treatment modalities and prevent the occurrence of disease per se.

Although, various HLA B27 exhibit polymorphism, but not all serotypes predispose an individual to AS.^7^ Earlier an Indian study reported frequency of HLA B27 in North Indian population at 6%.^8^ However, reports on the prevalence of HLA B27 in Pakistani population are scarce. Zafar et al. have reported the prevalence of HLA B27 at 5.5% in healthy kidney donors, from southern region of Pakistan.^9^ In our study cohort nearly 85% patients had positive HLA B27 gene. Whether or not the presence of HLA B27 gene in our AS patients, is the geographical distribution of HLA B27 gene in the general population of our region is not yet known. It has been reported that HLA B27 is present in approximately 6-8% of the normal population in Indian sub-continent. A study from Qatar reported positive HLA B27 in 61% of AS patients of Asian descent (Pakistanis, Indians, Iranian).^10^

It is plausible that the high number of Pathan patients in this study reflect a genetic pre-disposition. This is especially likely as inter-marriages within the family are a strictly observed norm in this ethnic group. Specific dietary habits with very high meat intake, high altitude and extremely cold weather are other factors specific to this group. They also suffer from scarcity of safe potable water, as well as water for personal hygiene. It is likely that infections are more common for these reasons. Apart from the association between HLA B27 and AS, microbial infections have been proposed as a predisposing factor for AS. A Finnish study identified that the surface protein of Yersinia and Salmonella share homology with HLA B27.^11^ As we know, Salmonella and other gut infections are very common in our part of the world; this may be one of the triggers to develop AS in a predisposed group of people.

A lag time of several years may pass between onset of symptoms and the diagnosis being established. An average of 8 to 11 years of delay between the onset of disease symptoms and diagnosis of AS has been reported.^12^ The same is reflected in this study as well. This delay is most likely due to low awareness among non-rheumatologists and general practitioners about AS, as chronic back pain is very common. It requires clinical experience to distinguish between inflammatory and mechanical backache. Pakistan being a country with poor health care resources, patients may ignore mild disease symptoms for years, until significant disability compels them to seek medical advice. Further, the inflammatory markers are not invariably elevated and the radiographic appearances of sacroilitis are a late feature of the disease. Thus a high index of suspicion is required for early diagnosis. It has been reported that the HLA B27 negative individuals had a higher average age at disease onset, compared to those with HLA B27 positive AS.^13^ However, no such association is evident in our study, perhaps due to small number of patients and the majority being HLA B27 positive. In addition, the general HLA B27 prevalence in the local population is not precisely known. The predilection of the disease in Pathan ethnicity, has not been reported before.

Apart from axial disease, peripheral joint involvement is frequently seen in AS. Many of these patients develop peripheral joint arthritis during the course of disease, often involving the knee joints. An increased frequency of peripheral arthritis has been seen in Indian patients with a reported peripheral joint involvement in upto 65% of AS patients.^14^ Our patients however, had involvement of peripheral joints later in the course of their disease. Extra-articular features form a major part of disease spectrum in all variants of SpA. The commonest being uveitis, with an increased prevalence in HLA B27 positive patients.^15^ Other extra-articular features include, symptomatic Inflammatory bowel disease (IBD) in 10% of patients, while asymptomatic colonic mucosal ulceration has been reported in 50-60% of AS patients.^16^,^17^ Most of our patients had constitutional symptoms of fatigue and fever with only a few having eye or genito-urinary symptoms. Our patients had no associated co-morbidity, probably on account of their relatively young age.

Limitations in physical functioning, including self-care, mobility, etc, tend to increase with duration of disease of most chronic musculoskeletal diseases. In AS, these have largely been attributed to limited spinal mobility. It has been suggested that the damage in different skeletal regions impact functioning over the duration of AS. Patients with AS can develop vertebral fractures even with minimal trauma. The life time risk of vertebral fractures is more in these patients compared to the general population.^18^,^19^

Tumor Necrosis Factor (TNF) appears to be a key factor in the inflammatory process of AS, thus several placebo controlled trials have demonstrated significant and sustained efficacy of TNF inhibitor in AS.^20^,^21^ The management guidelines based on available evidence suggest no effectiveness for Methotrexate or Sulphasalazine in axial disease.^22^ Our experience is contrary to this, as cost constraints compel as to use these agents rather than TNF inhibitors with good clinical response.

This study represents a small, but first systematic attempt to collate estimates of AS burden in our region. Extensive literature search revealed no single study reflecting the disease spectrum of AS in our country. It is apparent that the continuing conduct of epidemiological studies of AS prevalence is of great importance, particularly as diagnostic capabilities improve, earlier diagnosis might potentially reduce the crippling effects of this disease.

## CONCLUSION

The demographic features of AS are same as those reported in earlier studies across the globe. Our study provides some clues to the relative role of genetics and environmental factors in the development of AS in our geographical area. The plausible factors responsible for this suspected racial predisposition need to be studied. An infectious trigger, accounting for the pathogenesis of this disease, remains an intriguing hypothesis.
